# High N_2_ selectivity in selective catalytic reduction of NO with NH_3_ over Mn/Ti–Zr catalysts

**DOI:** 10.1039/c8ra00336j

**Published:** 2018-04-03

**Authors:** Bolin Zhang, Shengen Zhang, Bo Liu, Hanlin Shen, Lin Li

**Affiliations:** Institute for Advanced Materials and Technology, University of Science and Technology Beijing Beijing 100083 P. R. China zhangshengen@mater.ustb.edu.cn

## Abstract

A series of Mn-based catalysts were prepared by a wet impregnation method for the selective catalytic reduction (SCR) of NO with NH_3_. The Mn/Ti–Zr catalyst had more surface area, Lewis acid sites, and Mn^4+^ on its surface, and showed excellent activity and high N_2_ selectivity in a wide temperature range. NH_3_ and NO oxidation was investigated to gain insight into NO reduction and N_2_O formation. The formation of N_2_O was primarily dominated by the reaction of NO with NH_3_ in the presence of O_2_*via* the Eley–Rideal mechanism. An intimate synergistic effect existed between the Mn-based and the Ti–Zr support. It was demonstrated that the Ti–Zr support greatly promoted the catalytic performance of Mn-based catalysts.

## Abstract 2

The Ti–Zr support greatly promotes the catalytic performance of Mn-based catalysts.

## Graphical abstract

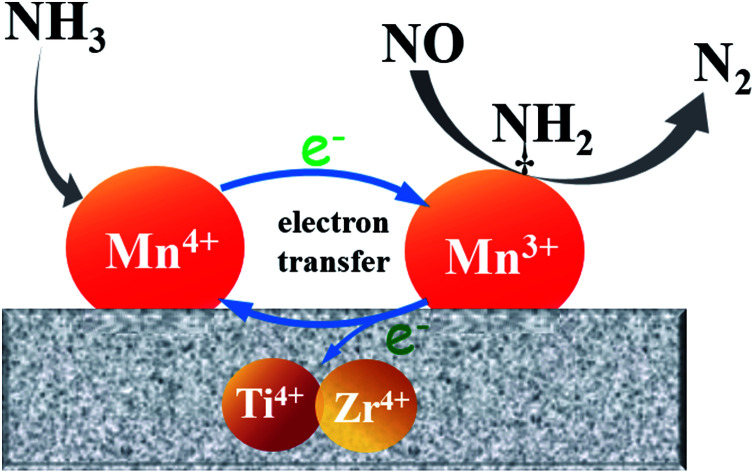

## 1. Introduction

According to the *BP Statistical Review of World Energy* June 2017, oil and coal remain the world's leading fuels, contributing to 61.4% of global energy consumption in 2016. The combustion of fossil fuels produces numerous nitrogen oxides, which has resulted in a series of environmental issues. The technique of selective catalytic reduction (SCR) of NO_*x*_ with NH_3_ (NH_3_-SCR) based on the V_2_O_5_–WO_3_(MoO_3_)/TiO_2_ catalyst has played a pivotal role in alleviating these issues since the 1970s.^1^ Nevertheless, 5.519 megatons of NO_*x*_ were emitted by coal-fired plants in China in 2015, with the low removal rate of 50.4%.^2^ Considering the advantages of energy conservation and long service time, low-temperature NH_3_-SCR is recognized as a promising candidate to optimize this technique and solve some of the critical problems, including the low removal rate and toxicity of vanadium species.

Mn-based catalysts, such as MnO_*x*_/TiO_2_,^3–7^ MnO_*x*_–CeO_2_,^8–11^ Mn–ZrO_2_,^12^ Fe–Mn/TiO_2_ (ref. 13) and Mn/Ce–ZrO_2_,^14^ show a relatively high activity at low temperature.^15^ Moreover, Mn could improve the performance of V_2_O_5_/TiO_2_ catalyst.^16^ However, most of previous work placed emphasis on the NO conversion. The overall performance has yet to be improved further, especially related to N_2_ selectivity and SO_2_ resistance. The formation of N_2_O occurs simultaneously during the SCR process over Mn-based catalysts.^10^ Moreover, the production of N_2_O goes up sharply along with the reaction temperature. This restricts its operation window and even its industrial application.

Some studies have been made to investigate the mechanism of N_2_O generation with the aim to improve N_2_ selectivity. For instance, aggregated MnO_*x*_ species accelerated the formation of N_2_O.^17^ MnO_*x*_ species supported on Fe–Ti spinel exhibits a good N_2_ selectivity for the special construction of Fe–Ti spinel.^18^ N_2_O formation over MnO_*x*_/TiO_2_ catalysts can be suppressed by doping with transition elements.^19^ The mechanism of N_2_O formation is not evident and the method to improve N_2_ selectivity, or in other words, to inhibit N_2_O generation, is insufficiently explained. In summary, Mn-based catalysts are potential candidates for low-temperature SCR and further efforts are needed to improve the overall performance. In this study, a MnO_*x*_/Ti–Zr catalyst was prepared by co-precipitation and impregnation methods. Compared to the MnO_*x*_/Ti and MnO_*x*_/Zr catalysts, the MnO_*x*_/Ti–Zr catalyst showed a higher NO conversion, N_2_ selectivity and a wider operation window. In addition, the mechanism of N_2_O formation was investigated.

## 2. Experimental

### 2.1. Catalyst preparation

The Ti–Zr support was synthesized by co-precipitation method using titanium sulfate (Ti(SO_4_)_2_, CP) and zirconium nitrate (Zr(NO_3_)_4_·5H_2_O, AP) as precursors and ammonium hydroxide (NH_3_·H_2_O, 25 wt%) as precipitator. Specified amounts of titanium sulfate and zirconium nitrate were dissolved in deionized water, and the mixture was added to a 12.5 wt% ammonium hydroxide solution dropwise under vigorous magnetic stirring. The resulting precipitate was filtered under vacuum, washed by deionized water 3 times, dried for 6 h at 90 °C. The obtained support was mainly composed of hydroxides. The molar ratio of Ti/Zr was set as 1 : 1. Ti and Zr supports were prepared following the same precipitation method.

The Mn/Ti–Zr catalyst was prepared by a wet impregnation method. Manganese nitrate (Mn(NO_3_)_2_·4H_2_O, CP) was dissolved in an appropriate amount of deionized water. Ti–Zr support was added to the solution under magnetic stirring for 30 min and treated by ultrasonic wave for another 30 min. The excess water was evaporated at 70 °C. Finally, the specimen was dried for 6 h at 90 °C and calcined under air for 2 h at 500 °C. The Mn/Ti and Mn/Zr catalysts were prepared following the same procedure. The atomic ratios of Mn/Ti, Mn/Zr and Mn/(Ti + Zr) were set as 3 : 10.

### 2.2. Catalytic activity measurements

The catalytic activity measurements of all specimens were carried out in a fixed-bed quartz tube reactor with 0.8 cm internal diameter at atmospheric pressure. The volume of the evaluated catalysts with 60–100 mesh was 0.5 cm^3^ and the corresponding mass was ∼350 milligram. The components of the simulated flue gas, with a total flow rate of 300 ml min^−1^ and gas hourly space velocity (GHSV) of 36 000 h^−1^ controlled by mass flow meters, was composed of 600 ppm NO, 600 ppm NH_3_, 5% O_2_ and N_2_ balance. The concentrations of NO, NO_2_, N_2_O, and O_2_ in the outlet flue gas were measured by an infrared gas analyzer paired up with a professional gas conditioner (Madur Photon & PGD-100, Austria). The concentrations of the feed gas were determined before measuring catalytic activity. The reaction temperature was increased from 100 to 340 °C and sampled at an interval of 20 °C. The NO conversion and N_2_ selectivity were calculated in terms of the following formulas:^9,20^
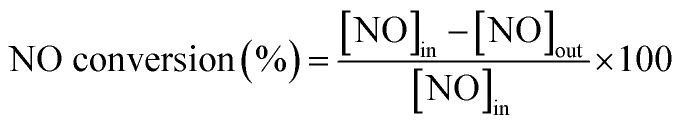

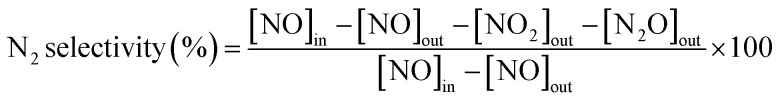
where [NO]_in_, [NO]_out_, [NO_2_]_out_ and [N_2_O]_out_ indicate the inlet and outlet concentration of NO, NO_2_ and N_2_O, respectively. All data were collected under the steady state condition.

The reaction rates normalized by the surface area were calculated with the following formula:^21^
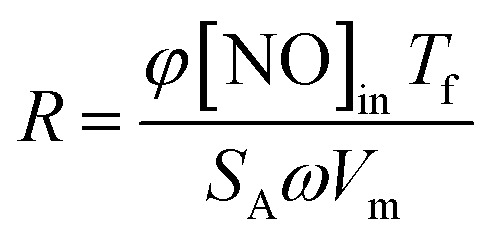
where *φ* and *T*_f_ are the NO conversion (%) and total flow rate (ml s^−1^). *S*_A_, *ω* and *V*_m_ are the specific surface area (m^2^ g^−1^), catalyst weight (g) and molar volume of gas (22.4 ml mmol^−1^).

### 2.3. Catalyst characterization

The powder X-ray diffraction (XRD) patterns of the samples were recorded on a Rigaku Ultima IV diffractometer (Japan) with Cu Kα radiation (*λ* = 1.5406 Å, 40 kV, 40 mA) by a sampling interval of 0.02 degree. The specific surface area, pore volume and average pore diameter of the prepared catalysts were determined from the nitrogen adsorption–desorption isotherms at liquid nitrogen temperature (77 K) by a Quadrasorb SI automated surface area and pore size analyzer (Quantachrome, USA). The Fourier transform infrared (FT-IR) spectra were recorded at room temperature on a FTIR-8400s Fourier transform infrared spectrophotometer (Shimadzu, Japan). Powder materials mixed with KBr were tableted into small rounds of 0.2–0.3 mm thickness. The contribution of KBr to the sample spectra was reduced by referring and normalizing to the spectrum of KBr.

The X-ray photoelectron spectroscopy (XPS) experiments were carried out on an EscaLab 250Xi ThermoFisher Scientific (USA) X-ray photoelectron spectrometer system equipped with a monochromated Al Kα (150 W) as a X-ray source. High resolution scans of binding energies were measured for C 1s, O 1s, Mn 2p, Ti 2p, and Zr 3d with the pass energy of 20 eV and an energy step size of 0.05 eV. The binding energies were modified by referencing to the C 1s binding energy of 284.8 eV. The O 1s and Mn 2p peak were deconvoluted optimally into several sub-bands by the Gaussian–Lorentzian function with the correlation coefficients (*r*^2^) above 0.99 referring to the overlapped peak.

## 3. Results and discussion

### 3.1. NH_3_-SCR activity

**Fig. 1:**
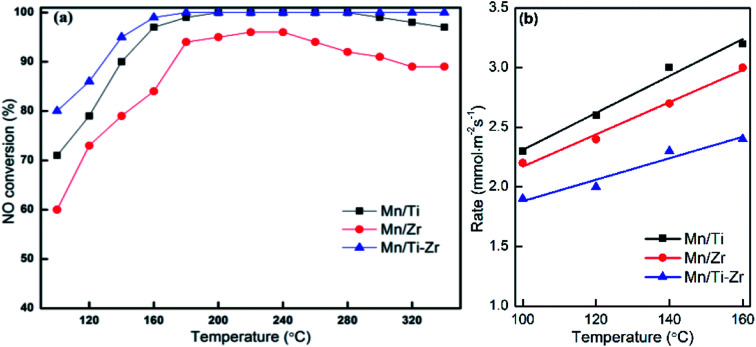
(a) NO conversion and (b) normalized reaction rates for different catalysts. Reaction conditions: [NO] = 600 ppm, [NH_3_] = 600 ppm, [O_2_] = 5%, N_2_ balance and GHSV = 36 000 h^−1^.

Fig. 1a shows the NO conversion of different catalysts. NO conversion increased overall with the increasing of temperature over Mn/Ti, Mn/Zr and Mn/Ti–Zr catalysts. The Mn/Ti–Zr catalyst showed the highest NO conversion in the investigated temperature range and the Mn/Zr catalyst showed a relatively lower conversion. Especially, excellent NO conversion of nearly 100% was obtained over both Mn/Ti–Zr and Mn/Ti catalysts in the temperature range of 180 to 280 °C. This result indicates that all of NO were involved in the catalytic reaction at 180 to 280 °C, and there were enough active sites for NO on the Mn/Ti–Zr and Mn/Ti. Fig. 1b presents the normalized reaction rates of different catalysts at 100 to 160 °C. The reaction rates were similar with the reported catalysts.^22^ Since of the high specific surface area (Table 1), the Mn/Ti–Zr catalyst obtained a low reaction rate. The result could indicate an equilibrium of reaction exists in the SCR reaction at this temperature.

**Table 1:** BET surface area, pore volume and average pore diameter of the samples

Samples	BET surface area (m^2^ g^−1^)	Pore volume (cm^3^ g^−1^)	Average pore diameter (nm)	Mole ratio of Mn/(Ti + Zr)
Mn/Ti	114	0.33	11.6	0.3
Mn/Zr	104	0.31	7.6	0.3
Mn/Ti–Zr	189	0.51	8.4	0.3

**Fig. 2:**
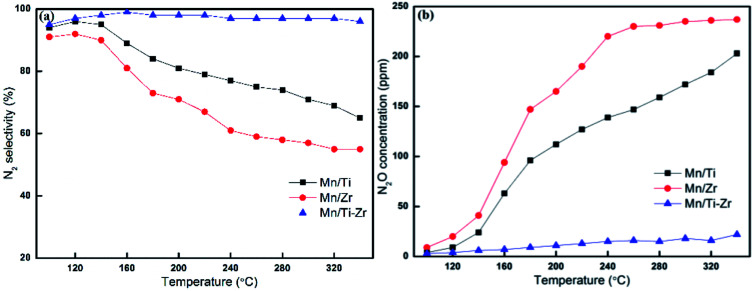
(a) N_2_ selectivity and (b) outlet concentration of N_2_O for different catalysts. Reaction conditions: [NO] = 600 ppm, [NH_3_] = 600 ppm, [O_2_] = 5%, N_2_ balance and GHSV = 36 000 h^−1^.

In general, Mn-based catalysts show a high activity when the reaction temperature is above 150 °C and however, the N_2_ selectivity decreases gradually along with the increasing of temperature.^23–26^Fig. 2a shows the N_2_ selectivity results of samples. Overall, the N_2_ selectivity of the Mn/Ti and Mn/Zr catalysts decreased with the increasing of temperature. The increase in N_2_ selectivity in the temperature range of 100 °C to 120 °C was attributed to the increasing in NO conversion. However, a high N_2_ selectivity (nearly 100%) was obtained above 160 °C for the Mn/Ti–Zr catalyst.

Fig. 2b shows the plot of N_2_O concentration, which could be a more appropriate parameter to describe the N_2_ selectivity, as a function of temperature. It can be clearly seen that a small amount of N_2_O was produced over the Mn/Ti–Zr catalyst. Nevertheless, N_2_O was generated in large quantities over the Mn/Ti and Mn/Zr catalysts with the maximum of 203 ppm and 237 ppm at 340 °C, respectively. The result manifests that even though NO was involved in the catalytic reaction over Mn/Ti and Mn/Zr, the product was N_2_O and not N_2_. As mentioned above, it is a common issue that N_2_ selectivity decreases with the increasing of temperature for Mn-based catalysts. Referring to the high N_2_ selectivity of Mn/Ti–Zr catalysts, it can be deduced that the Ti–Zr composite support is in favor of suppressing the generation of N_2_O and improving the N_2_ selectivity of Mn-based.

**Fig. 3:**
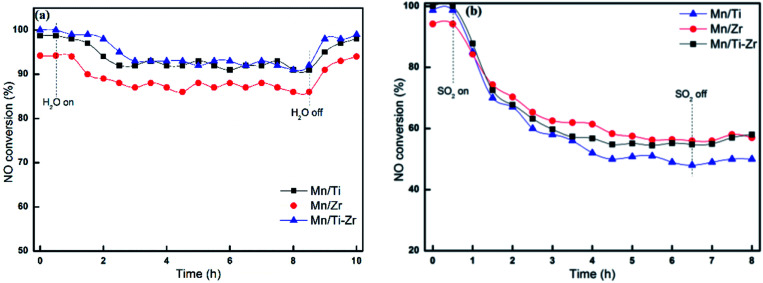
NO conversion for different catalysts in the presence of (a) 3 vol% H_2_O or (b) 50 ppm SO_2_ at 180 °C. Reaction conditions: [NO] = 600 ppm, [NH_3_] = 600 ppm, [O_2_] = 5%, N_2_ balance and GHSV = 36 000 h^−1^.

Fig. 3 reveals the durability of H_2_O and SO_2_ for different catalyst at 180 °C. The No conversion of all catalysts decreased slightly after the introducing of 3 vol% H_2_O (Fig. 3a). When 3 vol% H_2_O was cutting off, the NO conversion returned to the original level gradually. The result agrees with the previous publication that the competitive adsorption of H_2_O blocks the active sites. As shown in Fig. 3b, the NO conversion decreased sharply after 50 ppm SO_2_ was introduced at 180 °C. Moreover, when 50 ppm SO_2_ was removed, the catalytic activities cannot recover to the original level. The irreversible deactivation indicates that the deactivation by SO_2_ is ascribed to the sulfation of active sites and deposition of ammonium sulfates.^15^

### 3.2. NH_3_ and NO oxidation

**Fig. 4:**
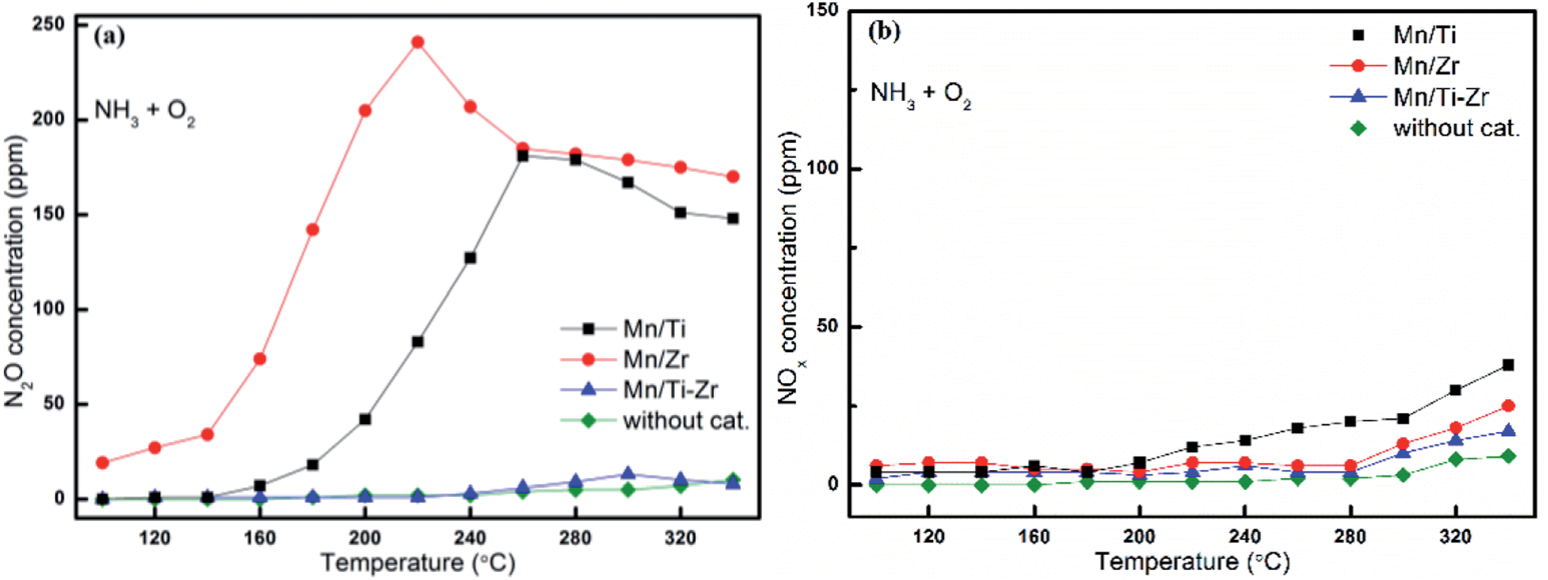
Oxidation of NH_3_ to (a) N_2_O and (b) NO_*x*_ (*x* = 1, 2) over different catalysts. Reaction conditions: [NH_3_] = 600 ppm, [O_2_] = 5%, N_2_ balance and GHSV = 36 000 h^−1^.

In order to investigate the origin of N_2_O and the catalytic oxidation activity of NH_3_ and NO, separate NH_3_ and NO oxidation experiments were carried out over Mn/Ti, Mn/Zr and Mn/Ti–Zr catalysts. The comparative experiments were conducted with an empty quartz tube as blank control group. Fig. 4 presents the results of NH_3_ and NO oxidation. As shown in Fig. 4a, only small quantities of N_2_O were detected over the Mn/Ti–Zr catalyst, which was nearly the same result as that achieved for the blank control group. The result indicated that the Mn/Ti–Zr catalyst could hardly catalyze NH_3_ to N_2_O. The Mn/Zr catalyst showed the best catalytic oxidation activity of NH_3_ to N_2_O, following with the Mn/Ti catalyst. These results agreed with the N_2_ selectivity results shown in Fig. 2. Furthermore, there was a downtrend of N_2_O generation over the Mn/Ti, Mn/Zr and Mn/Ti–Zr catalysts at 260, 220, and 300 °C, respectively. Referring to Fig. 2b, a different trend was observed with the results depicted in Fig. 4a. As shown in Fig. 2b, the concentration of N_2_O increased with the increase in temperature from 100 °C to 340 °C. Therefore, it can be concluded that the direct oxidation of NH_3_ is just one of the origins of N_2_O.

As shown in Fig. 4b, the concentrations of NO_*x*_ (*x* = 1, 2) originated from NH_3_ oxidation were far below the concentrations of N_2_O formed from NH_3_ oxidation, but increased slowly with the increasing temperature. NO could be oxidized to NO_2_ under oxygen atmosphere with a reaction equilibrium as shown in following equation:

1 2NO + O_2_ → 2NO_2_

**Fig. 5:**
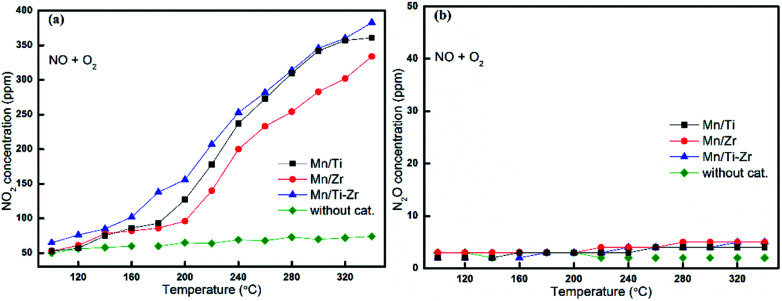
Formation of (a) NO_2_ and (b) N_2_O by the reaction of NO with O_2_ over different catalysts. Reaction conditions: [NO] = 600 ppm, [O_2_] = 5%, N_2_ balance and GHSV = 36 000 h^−1^.

It is well agreed that the SCR process could be accelerated by the generation of NO_2_ through the so-called Fast SCR process.^22,27^Fig. 5a shows the outlet concentrations of NO_2_ originated from NO oxidation for different catalysts, which represents the property of NO oxidation to NO_2_ as a function of temperature. It was observed that the concentrations of NO_2_ increased with the increasing temperature apparently.

The Mn/Ti–Zr catalyst obtained the highest NO_2_ concentration, followed by Mn/Ti and Mn/Zr in order. Comparing with Fig. 4b, it can be found that the favorable formation of NO_2_ mainly originated from the oxidation of NO. The results indicate that the Mn/Ti–Zr catalyst presented the best property of NO oxidation to NO_2_, which likely contributed to the Fast SCR process. Referring to the NO conversion as shown in Fig. 1a, there was a similar trend between NO conversion and NO_2_ formation. However, contrasting Fig. 1a with 5a, it can be found that the SCR reaction was not dominated by the Fast SCR process. Especially, there was a very low NO_2_ concentration but a high NO conversion in the temperature range of 100 °C to 160 °C. Thus, it could be deduced that the SCR process was primarily attributed to the Standard SCR. Fig. 5b shows the formation of N_2_O by a disproportionation reaction in the presence of O_2_. Hardly any N_2_O was detected in the outlet.

### 3.3. XRD and BET analysis

**Fig. 6:**
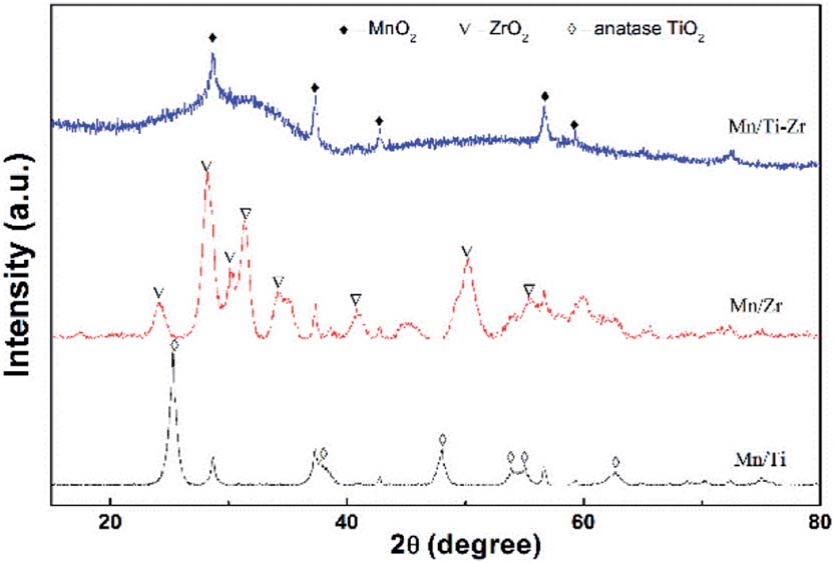
XRD patterns of the Mn/Ti, Mn/Zr and Mn/Ti–Zr catalysts.

Fig. 6 shows the XRD patterns of all samples. The diffraction peaks at 2*θ* = 28.7°, 37.4°, 42.8° and 56.7° assigned to the MnO_2_ species (PDF card No. 81-2261) were detected in all samples, since the atomic ratio of Mn/(Ti + Zr) with 3 : 10 is a heavy load. It can be speculated that the MnO_2_ species dispersed over the supports and the redundant MnO_2_ species was in crystal form. It has been widely reported that MnO_2_ exhibits excellent activity as compared to other MnO_*x*_ species.^28^ The diffraction peaks assigned to anatase TiO_2_ (PDF card No. 99-0008) and baddeleyite ZrO_2_ (PDF card No. 86-1449) were detected in the Mn/Ti and Mn/Zr samples, respectively. The results demonstrate that the support of TiO_2_ and ZrO_2_ were crystallized by calcined at 500 °C for 2 h.

However, strong amorphous peaks was detected for Mn/Ti–Zr sample, which was related to the amorphous structure of TiZrO_4_. The devitrification temperature of TiZrO_4_ is about 700 °C.^29^ The amorphous structure support provided a higher surface area and pore volume.^30^ The combination of titanium and zirconium enhanced the dispersion to some extent. This observation is consistent with the specific surface area and pore volume results (Table 1). In addition, the amorphous oxides could enhance the SCR activity owing to the strong interactions between Ti, Zr and Mn.^31,32^ In consideration of the catalytic activity, it is a reasonable inference that the support not only provided a sufficient surface area and pore volume, but was also involved in the SCR process.

BET surface area, pore volume and average pore diameter of the samples are given in Table 1. The decreasing order of the surface area and pore volume was Mn/Ti–Zr > Mn/Ti > Mn/Zr. This order was in accordance with that of the NO conversion. It agrees the generic studies that the surface area and pore volume are important to catalytic activity.

### 3.4. FT-IR analysis

**Fig. 7:**
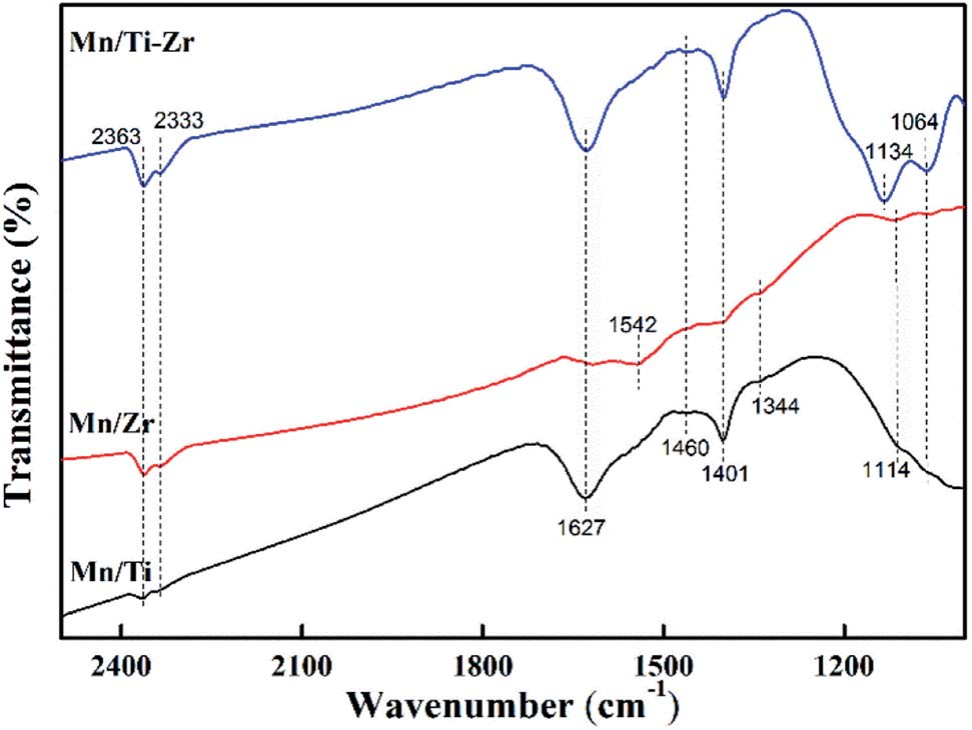
FT-IR spectra of the Mn/Ti, Mn/Zr and Mn/Ti–Zr catalysts.

The NH_3_ molecules adsorbed on the catalysts were analyzed by FT-IR spectroscopy at room temperature to obtain information about the acid sites and the behavior of ammonia on the surface. The prepared catalysts were exposed to the gas mixture of 2% NH_3_/N_2_ for 20 min and their FT-IR spectra were obtained (Fig. 7). The peaks at 1064, 1114, 1134 and 1627 cm^−1^ are assigned to the ammonia coordinated on the Lewis acid sites.^33,34^ The peaks at 1064 and 1627 cm^−1^ were observed for all samples and were stronger in the case of the Mn/Ti–Zr catalyst as compared to the Mn/Ti and Mn/Zr samples. A strong peak at 1134 cm^−1^ was observed for Mn/Ti–Zr and the weak peak at 1114 cm^−1^ was detected for Mn/Ti and Mn/Zr. It can be found that the peaks assigned to ammonia coordinated on the Lewis acid sites for Mn/Ti–Zr and Mn/Ti are stronger than that for Mn/Zr. In terms of the results, it can be deduced that the combination of Mn and Ti could promote formation of the Lewis acid sites. The peaks at around 1401 and 1460 cm^−1^ are assigned to the NH_4_^+^ of on the Brønsted acid sites.^9,14^ The peaks at 1344 and 1542 cm^−1^ detected for the Mn/Ti and Mn/Zr are related to the NH_3_ adsorbed on Brønsted acid sites.^19,35^ In addition, the peaks at 2363 and 2333 cm^−1^ are assigned to the anti-symmetric stretching vibration of CO_2_, which was adsorbed from the air.^36^

The results indicate that the Mn/Ti–Zr catalyst possesses more Lewis acid sites. The Mn/Ti catalyst showed strong peaks corresponding to Lewis acid sites and the Brønsted acid sites. Referring to the observed band of Mn/Zr, it is worth noting that peak at 1542 cm^−1^ was of a strong intensity, which indicated a high proportion of Brønsted acid sites on the Mn/Zr catalyst surface. It has been previously reported that Lewis acid sites are beneficial to NO conversion, which agreed with the result of activity measurements.^37^ Besides, the Brønsted acid sites are advantage for the adsorption of NH_3_.^38^ In fact, although gaseous NH_3_ could be adsorbed on both Lewis acid sites and Brønsted acid sites during the SCR process, it was the Lewis acid sites that mainly exerted a positive effect.

### 3.5. XPS analysis

X-ray photoelectron spectroscopy was carried out to get insight into the oxidation states and atomic concentrations of the surface layer of the catalysts. As listed in Table 2, the main element on the surface was oxygen with percentage concentrations of 66.81, 70.39, and 71.33 for the Mn/Ti, Mn/Zr and Mn/Ti–Zr, respectively. Moreover, the Mn/Zr catalyst obtained the highest concentration of surface Mn element (4.54%), which were 3.81% and 3.48% in the case of Mn/Ti–Zr and Mn–Ti, respectively.

**Table 2:** Surface atom contents in different samples

Sample	Surface element concentration (%)				Molar ratios	
	Mn	O	Ti	Zr	Mn^4+^/(Mn^4+^ + Mn^3+^)	O_α_/(O_α_ + O_β_)
Mn/Ti	3.48	66.81	29.72	—	0.62	0.21
Mn/Zr	4.54	70.39	—	25.06	0.49	0.42
Mn/Ti–Zr	3.81	71.33	11.07	13.8	0.67	0.40

**Fig. 8:**
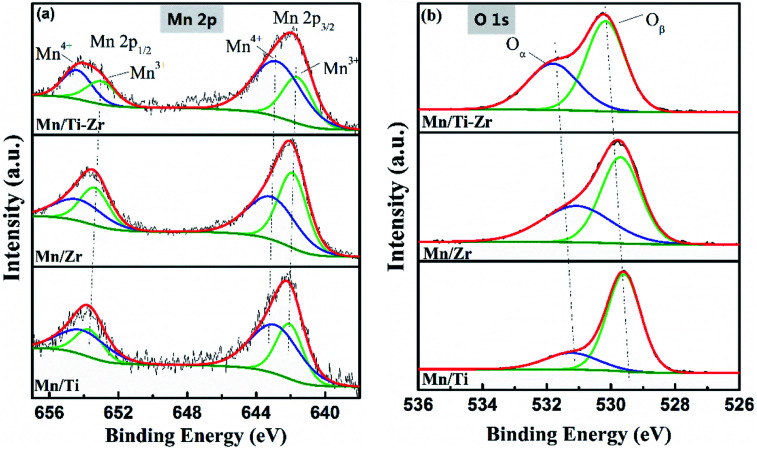
High resolution XPS spectra of (a) Mn 2p and (b) O 1s of Mn/Ti, Mn/Zr and Mn/Ti–Zr.

The high-resolution XPS spectra of Mn 2p and O 1s are shown in Fig. 8. Fig. 8a shows the high-resolution XPS spectra of Mn 2p (Mn 2p_1/2_ and Mn 2p_3/2_), which could be deconvoluted into two sets of double-peaks characterized Mn^3+^ and Mn^4+^ located in the ranges of 641.6–642.2 eV and 643.8–643.9 eV, repectively.^21,39^ The peaks assigned to Mn^4+^ were found at 642.9, 643.1, and 642.8 eV for the Mn/Ti, Mn/Zr and Mn/Ti–Zr catalysts, respectively. There was a shift to lower binding energy of Mn^4+^ over the Mn/Ti–Zr catalyst for the interaction between the manganese oxide and the support. It is reported that a lower binding energy indicates a more active state. This manifests the Mn^4+^ on the Mn/Ti–Zr promoting the catalytic reaction.^40–42^Table 2 presents the mole ratios of Mn^4+^/(Mn^4+^ + Mn^3+^). The Mn/Ti–Zr catalyst provided a highest ratio of Mn^4+^ species, followed by that of the Mn/Ti and Mn/Zr catalysts in descending order. The high ratio of Mn^4+^ may be attributed to the Ti component of the support. Referring to the FT-IR results, it was found that Mn/Zr–Ti and Mn/Ti samples have more Lewis acid sites and high ratios of Mn^4+^ simultaneously. Therefore, it is a reasonable speculation that the Mn^4+^ provides more Lewis acid sites.

As is well known, high concentration of surface Mn^4+^ is conducive to the catalytic oxidation ability and enhances the NH_3_-SCR activity.^34^ This result is in accordance with the catalytic activity, as shown in Fig. 1a. The superior NO conversion of the Mn/Ti–Zr and Mn/Ti catalysts could be attributed to the high concentration of Mn^4+^. Mn species have an intimate interaction between Mn and Ti–Zr, which allows more electronic transfer between Mn, Ti and Zr.^43,44^

The O 1s XPS spectra were deconvoluted into two sub-bands as shown in Fig. 8b. The higher binding energy of 531.0–531.8 eV are assigned to chemisorbed oxygen (defect-oxygen of O_2_^2−^/O^−^ and hydroxyl-like group of OH^−^) and the lower binding energy of 529.6–530.2 eV are assigned to surface lattice oxygen (O^2−^), donated as O_α_ and O_β_, respectively.^45,46^ The O_α_/(O_α_ + O_β_) ratios are given in Table 2. The Mn/Ti catalyst obtained the lowest O_α_ ratio and, on the contrary, showed a high ratio of O_β_, which could be attributed to the high crystallization of MnO_2_ and anatase TiO_2_. These results were consistent with those obtained from the XRD profiles, and the previous articles that stated that O_β_ was mainly derived from Ti–O of anatase TiO_2_ and O_α_ originated from the OH^−^ bonded with the metal cations of Mn, Ti, and Zr on the surface.^47^ The relative concentration ratio of O_α_ of Mn/Zr and Mn/Ti–Zr was twice that of the Mn/Ti. It was speculated that the Zr component of the support enhanced the formation of chemisorbed labile oxygen, which demonstrated a higher activity than lattice oxygen for the superior mobility. This facilitated the oxidation of NO to NO_2_ and consequently, increased the NH_3_-SCR performance.^48,49^ Moreover, there is a shift to higher binding energies for Mn/Ti–Zr samples, which could be ascribed to the interaction between the manganese oxide and Ti–Zr support.

### 3.6. Mechanism discussion

Based on the above analysis, a mechanism of NH_3_-SCR process and N_2_O formation were proposed to address the main reaction. It is widely reported that NO is mainly abated in two ways as follows:^50,51^

2 4NH_3_ + 4NO + O_2_ → 4N_2_ + 6H_2_O

3 4NH_3_ + 2NO + 2NO_2_ → 4N_2_ + 6H_2_O

As mentioned in Subsection 3.2, the generation of NO_2_ is dominated by reaction (1) and, however, the concentration of NO_2_ is very low in the temperature range of 100 °C to 160 °C (Fig. 5a). Thus, the NO reduction is dominated by reaction (2) and not reaction (3) in the temperature range of 100 °C to 160 °C. In fact, the Gibbs free energy for reaction (2) (Δ*G*^0^_298_ = −1651 kJ mol^−1^) is far below that of reaction (1) (Δ*G*^0^_298_ = −70 kJ mol^−1^).^15^ In addition, reaction (3) is limited by reaction (1). Therefore, it can be concluded that the reduction of NO was dominated by reaction (2) in the investigated temperature range.

**Fig. 9:**
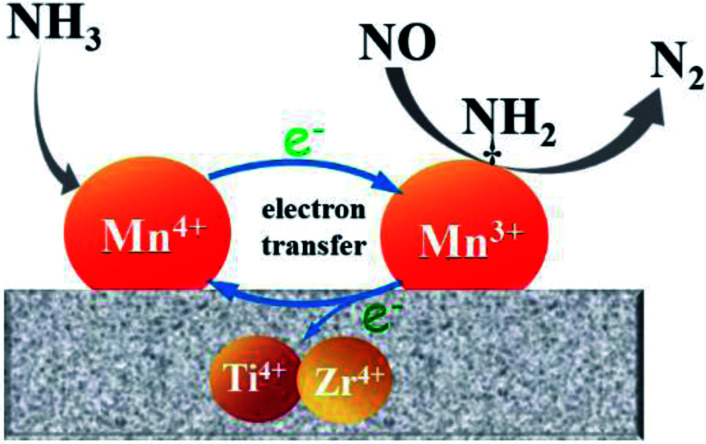
Schematic illustration of the redox cycle and NO reduction over the Mn/Ti–Zr catalyst.

On the basis of above analysis, the Mn/Ti–Zr catalyst showed the excellent NO conversion should be ascribed to the high concentration of Mn^4+^ and an intimated interaction between Mn and Ti–Zr, which allows more electronic transfer between Mn, Ti and Zr. A redox mechanism of NO reduction *via* the Eley–Rideal mechanism over the Mn/Ti–Zr catalyst is proposed (Fig. 9). Ti^4+^ and Zr^4+^ can restore the Mn^3+^ to Mn^4+^. The intimate interaction facilitates the electronic transfer and accelerates the circulation of Mn^4+^ and Mn^3+^ redox couple, which promotes the NH_3_-SCR process.

N_2_O can be generated by several approaches, including the disproportionation reaction of NO, NH_3_ oxidation and the reaction of NO_*x*_ with NH_3_. The low N_2_O formation to should be ascribed to the synergistic effect between Mn species and Ti–Zr support as shown in Fig. 9. The disproportionation reaction of NO can be described as the following reaction:^52,53^

4 3NO → N_2_O + NO_2_

Referring to Fig. 5b, almost no N_2_O was generated by the disproportionation reaction of NO in the presence of O_2_. The result indicates that reaction (4) can barely happen when oxygen is fed into the flue gas.

In the presence of O_2_, NH_3_ oxidation leads to the undesired ammonia loss and decreases the N_2_ selectivity of the NH_3_-SCR process. The following reactions describe the NH_3_ oxidation process.^54–56^

5 4NH_3_ + 3O_2_ → 2N_2_ + 6H_2_O

6 4NH_3_ + 4O_2_ → 2N_2_O + 6H_2_O

7 4NH_3_ + 5O_2_ → 4NO + 6H_2_O

8 4NH_3_ + 7O_2_ → 4NO_2_ + 6H_2_O

Referring to Fig. 4, it is evident that NH_3_ oxidation is dominated by reaction (6), and the reaction (7) and (8) occurred difficultly in the SCR process. The Mn/Ti–Zr catalyst exhibited a low catalytic oxidation activity of NH_3_ to N_2_O, which also explains the high N_2_ selectivity. Analysing the total N atoms, reaction (5) should exist in the process. As mentioned in Subsection 3.2, it is evident by comparing Fig. 2b and 4a that the concentration of N_2_O increased in the NH_3_-SCR process, while the concentration of N_2_O generated by reaction (6) decreased at high temperature. It can be concluded that the NH_3_ oxidation is just one of the origin of N_2_O. Referring to the previous studies, more N_2_O could be originated by the following reactions:^19,56,57^

9 4NH_3_ + 4NO + 3O_2_ → 4N_2_O + 6H_2_O

10 4NH_3_ + 4NO_2_ + O_2_ → 4N_2_O + 6H_2_O

Similar to the Fast SCR, reaction (10) is limited by the generation of NO_2_ and is not the main reaction for N_2_O formation. In conclusion, both reaction (6) and (9) could generated N_2_O but reaction (9) dominated the generation of N_2_O. It is reported that N_2_O is mainly generated from reaction (9)*via* the Eley–Rideal mechanism and not the Langmuir–Hinshelwood mechanism.^13,53^ In addition, the Langmuir–Hinshelwood mechanism is suppressed at high temperature and the Eley–Rideal mechanism dominates the NH_3_-SCR process subsequently.^58^ In a similar manner, N_2_O formation is enhanced *via* the Eley–Rideal mechanism at high temperature. This is consistent with the results shown in Fig. 2b. Based on the analysis above, a mechanism of N_2_O formation is proposed as follows:^37^

11 NH_3_(ad) + Mn^4+^ = O → NH_2_(ad) + Mn^3+^–OH

12 NH_2_(ad) + Mn^4+^ = O → NH(ad) + Mn^3+^–OH

13 NH(ad) + NO(g) + Mn^4+^ = O → N_2_O(g) + Mn^3+^–OH

where (ad) and (g) indicate the adsorbed and gaseous molecules, respectively. Adsorbed NH_2_ can react with gaseous NO to form N_2_ and H_2_O. However, if a hydrogen was deprived further from NH_2_, N_2_O will be generated by the reaction between NH and NO.

## 4. Conclusion

Mn/Ti–Zr, Mn/Zr and Mn/Ti catalysts, prepared by a wet impregnation method, showed good NO conversion in the NH_3_-SCR in a wide temperature range. The Mn/Ti–Zr catalyst showed a high N_2_ selectivity and suppressed the oxidation of NH_3_ to N_2_O. The formation of N_2_O was primarily dominated by the reaction of NO with NH_3_ in the presence of O_2_*via* the Eley–Rideal mechanism. The Mn/Ti–Zr catalyst possessed more special surface area, Lewis acid sites and Mn^4+^ on catalyst's surface, which synergistically enhanced the catalytic activity and N_2_ selectivity. It was also found that the Ti–Zr support greatly promoted the performance of Mn-based catalysts in the NH_3_-SCR, especially the N_2_ selectivity. There was an intimate synergistic effect between the Mn species and the Ti–Zr support. In order to improve the catalytic performance, further research is required with a focus on not only the active component but also the support. Moreover, more researches will be done to clarify the reaction mechanism and the synergistic effect further.

## Conflicts of interest

There are no conflicts to declare.

## Supplementary Material

## References

[cit1] Radojevic M. (1998). Environ. Pollut..

[cit2] M.o.E.P.o.t.P.s.R.o. China , Annual Statistic Report on Environment in China, 2015, Ministry of Environmental Protection of the People's Republic of China, Beijing, 2017

[cit3] Guo R., Li M., Sun P., Liu S., Wang S., Pan W., Liu S., Liu J., Sun X. (2017). RSC Adv..

[cit4] Li B., Xiong S., Liao Y., Xiao X., Huang N., Geng Y., Zou S., Yang S. (2016). J. Phys. Chem. C.

[cit5] Park E., Kim M., Jung H., Chin S., Jurng J. (2013). ACS Catal..

[cit6] Wang X., Li X., Zhao Q., Sun W., Tade M., Liu S. (2016). Chem. Eng. J..

[cit7] Pappas D. K., Boningari T., Boolchand P., Smirniotis P. G. (2016). J. Catal..

[cit8] Yan D., Yu Y., Huang X., Liu S., Liu Y. (2016). J. Fuel Chem. Technol..

[cit9] Xiao X., Sheng Z., Yang L., Dong F. (2016). Catal. Sci. Technol..

[cit10] Liu Z., Yi Y., Zhang S., Zhu T., Zhu J., Wang J. (2013). Catal. Today.

[cit11] Qi G., Yang R. T., Chang R. (2004). Appl. Catal., B.

[cit12] Liu Z., Liu Y., Li Y., Su H., Ma L. (2016). Chem. Eng. J..

[cit13] Jiang B., Deng B., Zhang Z., Wu Z., Tang X., Yao S., Lu H. (2014). J. Phys. Chem. C.

[cit14] Shen B., Zhang X., Ma H., Yao Y., Liu T. (2013). J. Environ. Sci..

[cit15] Zhang S., Zhang B., Liu B., Sun S. (2017). RSC Adv..

[cit16] Liu Z., Li Y., Zhu T., Su H., Zhu J. (2014). Ind. Eng. Chem. Res..

[cit17] Kijlstra W. S., Poels E. K., Bliek A., Weckhuysen B. M., Schoonheydt R. A. (1997). J. Phys. Chem. B.

[cit18] Yang S., Qi F., Xiong S., Dang H., Liao Y., Wong P. K., Li J. (2016). Appl. Catal., B.

[cit19] Niu Y., Shang T., Hui S., Zhang X., Lei Y., Lv Y., Wang S. (2016). Fuel.

[cit20] Xiong Y., Tang C., Yao X., Zhang L., Li L., Wang X., Deng Y., Gao F., Dong L. (2015). Appl. Catal., A.

[cit21] France L. J., Yang Q., Li W., Chen Z., Guang J., Guo D., Wang L., Li X. (2017). Appl. Catal., B.

[cit22] Huang L., Zha K., Namuangruk S., Junkaew A., Zhao X., Li H., Shi L., Zhang D. (2016). Catal. Sci. Technol..

[cit23] Guo R., Wang Q., Pan W., Zhen W., Chen Q., Ding H., Yang N., Lu C. (2014). Appl. Surf. Sci..

[cit24] Shi Y., Chen S., Sun H., Shu Y., Quan X. (2013). Catal. Commun..

[cit25] Lu X., Song C., Jia S., Tong Z., Tang X., Teng Y. (2015). Chem. Eng. J..

[cit26] Liu Z., Zhu J., Li J., Ma L., Woo S. I. (2014). ACS Appl. Mater. Interfaces.

[cit27] Opitz B., Bendrich M., Drochner A., Vogel H., Hayes R. E., Forbes J. F., Votsmeier M. (2015). Chem. Eng. J..

[cit28] Kapteijn F., Singoredjo L., Andreini A., Moulijn J. A. (1994). Appl. Catal., B.

[cit29] Wei H., Yan X., Li X., He S., Sun C. (2013). J. Hazard. Mater..

[cit30] Boningari T., Ettireddy P. R., Somogyvari A., Liu Y., Vorontsov A., McDonald C. A., Smirniotis P. G. (2015). J. Catal..

[cit31] Liu J., Li X., Zhao Q., Ke J., Xiao H., Lv X., Liu S., Tadé M., Wang S. (2017). Appl. Catal., B.

[cit32] Li P., Xin Y., Li Q., Wang Z., Zhang Z., Zheng L. (2012). Environ. Sci. Technol..

[cit33] Li X., Li J., Peng Y., Chang H., Zhang T., Zhao S., Si W., Hao J. (2016). Appl. Catal., B.

[cit34] Thirupathi B., Smirniotis P. G. (2012). J. Catal..

[cit35] Thirupathi B., Smirniotis P. G. (2011). Appl. Catal., B.

[cit36] Kantcheva M., Milanova M., Mametsheripov S. (2012). Catal. Today.

[cit37] Yang S., Xiong S., Liao Y., Xiao X., Qi F., Peng Y., Fu Y., Shan W., Li J. (2014). Environ. Sci. Technol..

[cit38] Maqbool M. S., Pullur A. K., Ha H. P. (2014). Appl. Catal., B.

[cit39] Stanciulescu M., Caravaggio G., Dobri A., Moir J., Burich R., Charland J. P., Bulsink P. (2012). Appl. Catal., B.

[cit40] Boxiong S., Yan Y., Jianhong C., Xiaopeng Z. (2013). Microporous Mesoporous Mater..

[cit41] Fang N., Guo J., Shu S., Luo H., Chu Y., Li J. (2017). Chem. Eng. J..

[cit42] Shen B., Wang F., Liu T. (2014). Powder Technol..

[cit43] Ettireddy P. R., Ettireddy N., Mamedov S., Boolchand P., Smirniotis P. G. (2007). Appl. Catal., B.

[cit44] Huang L., Hu X., Yuan S., Li H., Yan T., Shi L., Zhang D. (2017). Appl. Catal., B.

[cit45] Gao C., Shi J., Fan Z., Yu Y., Chen J., Li Z., Niu C. (2017). Fuel Process. Technol..

[cit46] Zuo J., Chen Z., Wang F., Yu Y., Wang L., Li X. (2014). Ind. Eng. Chem. Res..

[cit47] Zhang Z., Chen L., Li Z., Li P., Yuan F., Niu X., Zhu Y. (2016). Catal. Sci. Technol..

[cit48] Fan J., Ning P., Song Z., Liu X., Wang L., Wang J., Wang H., Long K., Zhang Q. (2018). Chem. Eng. J..

[cit49] Chen H., Xia Y., Huang H., Gan Y., Tao X., Liang C., Luo J., Fang R., Zhang J., Zhang W., Liu X. (2017). Chem. Eng. J..

[cit50] Chang H., Chen X., Li J., Ma L., Wang C., Liu C., Schwank J. W., Hao J. (2013). Environ. Sci. Technol..

[cit51] Stanciulescu M., Bulsink P., Caravaggio G., Nossova L., Burich R. (2014). Appl. Surf. Sci..

[cit52] Boxiong S., Hongqing M., Chuan H., Xiaopeng Z. (2014). Fuel Process. Technol..

[cit53] Tang X., Li J., Sun L., Hao J. (2010). Appl. Catal., B.

[cit54] Kim M. H., Park S. W. (2016). Catal. Commun..

[cit55] Pietrogiacomi D., Magliano A., Ciambelli P., Sannino D., Campa M. C., Indovina V. (2009). Appl. Catal., B.

[cit56] You Y., Chang H., Zhu T., Zhang T., Li X., Li J. (2017). Mol. Catal..

[cit57] Zhang D., Yang R. T. (2017). Appl. Catal., A.

[cit58] Yang S., Wang C., Li J., Yan N., Ma L., Chang H. (2011). Appl. Catal., B.

